# Supplementary material for: Complexome profiling of the Chlamydomonas psb28 mutant reveals TEF5 as an early PSII assembly factor
Source: Plant Cell. 2025 Mar 17;37(6):koaf055. doi: 10.1093/plcell/koaf055 (PMC12164590; doi:10.1093/plcell/koaf055)
Supplement: koaf055_Supplementary_Data [file koaf055_supplementary_data.zip › psb28_interactive.zip!/plots/intensity/qualityPlots/peptides/GPT3_Cre12.g503000.t1.2.html]

### Cre12.g503000.t1.2

Trivial name: GPT3  
SeqCoverage [median]: 5.30  
Mass: 54.55 kDa  
  
 

| Sequence | MissedCleavage | Mass [Da] | wtIntensity | muIntensity | Score |
| --- | --- | --- | --- | --- | --- |
| ADAALER | 0 | 744.38 | 0.00 | 1184.09 | 127.12 |
| SGRPNYEEELVPLGGVSGSK | 1 | 2074.03 | 892.38 | 10566.94 | 66.87 |
|  |  |  | 892.4 | 11751.0 |  |
